# Identification of Genetic Mutations in Cancer: Challenge and Opportunity in the New Era of Targeted Therapy

**DOI:** 10.3389/fonc.2019.00263

**Published:** 2019-04-16

**Authors:** Jing Jin, Xu Wu, Jianhua Yin, Mingxing Li, Jing Shen, Jing Li, Yueshui Zhao, Qijie Zhao, Jingbo Wu, Qinglian Wen, Chi Hin Cho, Tao Yi, Zhangang Xiao, Liping Qu

**Affiliations:** ^1^Department of Oncology, The Affiliated Hospital of Southwest Medical University, Southwest Medical University, Luzhou, China; ^2^Laboratory of Molecular Pharmacology, Department of Pharmacology, School of Pharmacy, Southwest Medical University, Luzhou, China; ^3^South Sichuan Institute of Translational Medicine, Luzhou, China; ^4^Department of Oncology and Hematology, Hospital (T.C.M) Affiliated to Southwest Medical University, Luzhou, China; ^5^School of Chinese Medicine, Hong Kong Baptist University, Hong Kong, China; ^6^College of Pharmacy, Chengdu University of Traditional Chinese Medicine, Chengdu, China

**Keywords:** targeted therapy, cyclin-dependent kinases 4/6, somatic mutation, resistance, EGFR, PD-1/PD-L1

## Abstract

The introduction of targeted therapy is the biggest success in the treatment of cancer in the past few decades. However, heterogeneous cancer is characterized by diverse molecular alterations as well as multiple clinical profiles. Specific genetic mutations in cancer therapy targets may increase drug sensitivity, or more frequently result in therapeutic resistance. In the past 3 years, several novel targeted therapies have been approved for cancer treatment, including drugs with new targets (i.e., anti-PD1/PDL1 therapies and CDK4/6 inhibitors), mutation targeting drugs (i.e., the EGFR T790M targeting osimertinib), drugs with multiple targets (i.e., the EGFR/HER2 dual inhibitor neratinib) and drug combinations (i.e., encorafenib/binimetinib and dabrafenib/trametinib). In this perspective, we focus on the most up-to-date knowledge of targeted therapy and describe how genetic mutations influence the sensitivity of targeted therapy, highlighting the challenges faced within this era of precision medicine. Moreover, the strategies that deal with mutation-driven resistance are further discussed. Advances in these areas would allow for more targeted and effective therapeutic options for cancer patients.

## Introduction

Targeted therapies usually present with high selectivity, target precisely to specific gene or protein, and exert a biological function with minimal side effects (1), which has distinguished them from most conventional non-specific chemotherapeutic drugs. Targeted therapy has thus been regarded as the biggest success in the treatment of cancer in the past few decades. Many novel promising agents have been experimentally designed and developed and are increasingly entering clinical evaluation. However, the frequently observed alterations in the drug targets have posed a big challenge to successful cancer treatment. Genetic mutations in cancer are resulted from both inherited and environmental factors. In a recent report, it is demonstrated that a large proportion of cancer-related mutations are due to randomized DNA replication errors (2). Notably, the mutations in cancer therapy targets can greatly affect drug sensitivity. Mutation-driven drug resistance is very common in cancer. The efficacy of targeted therapy is thus largely dependent on the mutation profile of tumors in patients. Accurate molecular and genetic profiling of tumor cells is becoming a routine practice before the introduction of targeted therapy in patients.

In recent years, great progress has been made in targeted therapy discovery. Notably, many new drugs are designed primarily based on specific genetic background. For instance, nearly 40–50% of metastatic cutaneous melanoma possess v-raf murine sarcoma viral oncogene homolog B1 (BRAF) mutations (3), and ~90% of these BRAF mutations are caused by substitution of glutamic acid for valine at codon 600 (V600E) (4). Two selective BRAF inhibitors vemurafenib and dabrafenib were approved for the treatment of patients with BRAF-V600E mutation, showing improved progression-free survival (5). In November 2018, the U.S. Food and Drug Administration (FDA) approved an inhibitor of tropomyosin receptor kinases (TRKs), larotrectinib, for treatment of any type of solid tumors with TRK gene fusion (6). This is the second targeted therapy approved not for specific cancer types but for any cancers with specific mutations. Targeted therapies are becoming more precise.

In this perspective, we focus on the updated knowledge of targeted therapy in the last 3 years and describe how genetic mutations influence sensitivity of targeted therapy, highlighting the challenges faced within this era of precision medicine. Moreover, the strategies dealing with mutation-driven resistance are further discussed.

## Influence of Genetic Mutation on Sensitivity of Targeted Therapy

It is well-acknowledged that mutations in therapeutic targets can increase or decrease drug sensitivity (Table 1). The main challenge of targeted therapy today is the identification of particular cancer mutations which affect efficacy of targeted therapies as well as the identification of a specific group of patients most likely or unlikely to respond to certain targeted therapies. Despite the great challenges, in the last 3 years, we have seen significant progress in targeted therapy (Table 2), largely owing to the rise of large-scale sequencing technology and big data analysis. Several novel targets, including the programmed death-1/programmed death-ligand 1 (PD1/PDL1) and cyclin-dependent kinases 4 and 6 (CDK4/6), have been validated, with several new targeted drugs being approved. Some newly approved drugs are directly designed to deal with some known activating mutations, such as the T790M mutation in epidermal growth factor receptor (EGFR). Moreover, many new findings have been added to our knowledge of how mutations influence targeted therapies [e.g., the inhibitors of human epidermal growth factor receptor 2 (HER2) and anaplastic lymphoma kinase (ALK)]. Here, based on the most updated research in the last 3 years, we summarize the recent advances of several targeted therapies.

**Table 1:** Therapeutic response of targeted therapy in mutant cancers.

**Drugs**	**Sensitivity**	**Target mutations**	**Cancer types**	**Reference**
Gefitinib	+	EGFR-L858R	Lung cancer	(7)
Erlotinib	+	EGFR-L858R	Lung cancer	(7)
Gefitinib	–	EGFR-T789M	Lung cancer	(8)
Osimertinib	+	EGFR-T790M	Lung cancer	(9)
Osimertinib	–	EGFR-L718Q	Lung cancer	(10)
Trastuzumab	–	HER2-A859T, -G776L	Lung cancer	(11)
Afatinib	+	HER2-p.Tyr772_Ala775dup	Lung cancer	(12)
Neratinib	–	HER2-T798I, -L869R	Breast cancer	(13)
Lapatinib	–	HER2-T798M	Breast cancer	(14)
Trastuzumab	–	HER2-T798M	Breast cancer	(14)
Neratinib	+	HER2-S310, -L755, -V777, -G778_P780dup, and -Y772_A775dup	Breast, cervical and biliary cancers	(15)
Crizotinib	–	ALK-C1156Y, -L1196M	Lung cancer	(16, 17)
Lorlatinib	–	ALK-L1198F	Lung cancer	(18)
2,4-Pyrimidinediamine derivative	–	EML4-ALK-C1156Y, -L1196M	Lung cancer	(19)
TAE684	–	EML4-ALK-L1152R	Lung cancer	(20)
Dabrafenib	+	BRAF-V600E	Melanoma	(5)

**Table 2:** Cancer targeted therapy approved by FDA in 2017 and 2018.

**Drugs**	**Targets**	**Cancer types**
Pembrolizumab (2017)	PD-1	Solid tumor with mismatch repair deficiency or microsatellite instability
Cemiplimab (2018)	PD-1	Squamous cell carcinoma
Durvalumab (2017)	PD-L1	Urothelial carcinoma
Avelumab (2017)	PD-L1	Merkel cell carcinoma, urothelial carcinoma
Brigatinib (2018)	ALK	ALK-positive NSCLC
Lorlatinib (2018)	ALK	ALK-positive NSCLC
Ribociclib (2017)	CDK4/6	Breast cancer
Abemaciclib (2017)	CDK4/6	Breast cancer
Niraparib (2017)	PARP	Ovarian cancer, peritoneal cancer
Dacomitinib (2018)	EGFR	NSCLC with EGFR exon 19 deletion or exon 21 L858R substitution mutations
Talazoparib (2018)	PARP	Breast cancer with germline BRCA mutations
Duvelisib (2018)	PI3Kδ, PI3Kγ	Chronic lymphocytic leukemia, small lymphocytic lymphoma
Larotrectinib (2018)	TRKs	Solid tumor with TRK gene fusion
Neratinib (2017)	EGFR/HER2	HER2-amplified breast cancer

### Anti-PD1/PDL1 Therapies

So far, there are a total of 6 anti-PD1/PDL1 therapies that have been approved by the FDA. Notably, in 2017, a PD1 antibody pembrolizumab was approved for the treatment of any solid tumor with a mismatch repair deficiency or a microsatellite instability. Monotherapy of PD1/PDL1 blockade has received great success in many types of cancers (21, 22). However, there are certain patients that are gradually developing resistance after an initial response (23). Mutation-driven resistance of anti-PD1/PDL1 therapies has recently been studied in a small number of cancer patients. Zaretsky et al. reported that mutations of JAK1/JAK2 led to the desensitization of cancer cells to IFN-γ and contributed to an acquired resistance of pembrolizumab in patients with melanoma (23). Moreover, in one resistant patient, a frame-shift deletion in exon 1 of the β-2-microglobulin was detected, which may result in the loss of expression of surface the MHC class I (23). More studies are advocated to explore the acquired resistance of immune checkpoint inhibitors.

### Resistance of CDK4/6 Inhibitors

Currently, three CDK 4/6 selective targeting inhibitors, palbociclib, ribociclib, and abemaciclib have been approved to treat breast cancer. CDK4/6 inhibitors are increasingly used in clinical settings, but patients eventually show disease progression and the major reasons remain unclear (24). Dysregulation of cyclin D1-CDK4/6-retinoblastoma (Rb) pathway has been implicated in hormone receptor positive (HR^+^) breast cancer and in chemotherapeutic drug-resistance. Rb is usually intact in HR^+^ breast cancer and is important for the efficacy of CDK4/6-inhibitors in the treatment of breast cancer (25). It is indicated that T47D cells that become resistant to CDK4/6 inhibitors, develop CCNE1 amplification or Rb1 loss (26). Moreover, the acquisition of multiple *de novo* somatic Rb1 mutations in metastatic breast cancer patients may result in the emergence of a resistance to CDK 4/6 inhibitors (24). Until now, there has been no report on CDK4/6 mutations in cancer patients and their effect on efficacy of CDK4/6 inhibitors.

### EGFR and Different Generation of Tyrosine Kinase Inhibitors (TKIs)

EGFR is a prevalent target in several human cancers, such as lung, breast, colorectal, thyroid, and melanoma cancer. In lung cancer, several generations of small-molecular inhibitors have been developed to target the EGFR tyrosine kinases (27), such as inhibitors gefitinib, erlotinib, osimertinib, and necitumumab. The EGFR mutation in non-small cell lung cancer (NSCLC) was first identified in 2004, and the major missense and deletion mutation of EGFR in NSCLC occurs in the tyrosine kinase-coding domain in exons 18–21 (28). The L858R mutation in the exon 21 and exon-19 frame deletion are the most commonly detected mutation types of EGFR, representing 50 and 40% of tumor patients, respectively (7). These two types of mutations are sensitive to EGFR tyrosine kinase inhibitors (TKIs) in NSCLC. The first-generation TKIs, gefitinib and erlotinib, have a high selective inhibitory activity against both wild-types and these sensitive mutant EGFR (29). Previous studies show that gefitinib and erlotinib are important for the first-line treatment of NSCLC patients with the sensitive EGFR mutations (30, 31). On the other hand, another mutation T790M, a secondary EGFR mutation emerging in NSCLC, can lead to the resistance of more than half of patients' TKIs treatment (32). Very recently, the third-generation TKI inhibitor osimertinib has been approved to effectively target to EGFR T790M mutation with a response rate of 61% in NSCLC, significantly extending the overall survival in patients with the T790M mutation (9). However, the further mutation of a residue in the P-loop (L718Q) has been found to cause resistance to osimertinib (10). Nevertheless, though diverse EGFR mutations are present, the overall survival of lung cancer patients is markedly improved with TKI therapy.

### HER2 and Its Inhibitors

In breast cancer, the overall HER2 mutation rate is ~1.6% (25 out of 1,499 patients). In a study by Bose et al., seven HER2 somatic mutations including G309A, D769H, D769Y, V777L, P780ins, V842I, and R896C, have been identified as activating mutations (33). Several patients with HER2 activating mutations are resistant to the reversible HER2 inhibitor lapatinib, but sensitive to the irreversible HER2 inhibitor neratinib. Neratinib as a dual inhibitor of HER2 and EGFR was approved by FDA in 2017. It has been shown that the HER2 L755S mutation results in an acquired resistance to lapatinib in breast cancer, which could be overcome by the neratinib (34). In another study, the HER2-T798I gatekeeper mutation in breast cancer patients with a AHER2-L869R mutation was identified as a mechanism of acquired resistance to neratinib (13). The trial of neratinib has also been conducted in colorectal cancer (CRC) patients. The HER2 gene amplification and mutation in CRC can lead to the resistance of EGFR-targeted therapies cetuximab and panitumumab (35, 36). A negative effect of neratinib monotherapy has recently been confirmed in 12 CRC patients with different tumors harboring HER2 mutations (15). There were no positive therapeutic response and the median PFS was only 1.8 months, indicating that monotherapy with neratinib is ineffective. The underlying mechanisms still require further investigations.

### ALK and Different Generation of ALK Inhibitors

ALK has long been identified as a therapeutic target in cancer. The first ALK inhibitor crizotinib was approved by the FDA in 2011 (37). Although most NSCLC patients respond to this drug, tumors become resistant after 1–2 years of treatment. Around 1/3 of crizotinib-resistant tumors harbor mutations within the ALK kinase domain. The most commonly observed mutations of L1196M and G1269A lead to a decreased affinity for crizotinib (38). Other ALK point mutations, such as L1152R, C1156Y, I1171T, F1174L, G1202R, and S1206Y, are also associated with crizotinib resistance (39). Another oncoprotein of fusion-type tyrosine kinase, the EML4-ALK, results from the inversion within the short arm of the human chromosome 2 in 4–5% of cases of NSCLC (40). Two mutations of EML4-ALK, C1156Y, and L1196M, confer a significant resistance to ALK inhibitors, such as crizotinib and PDD (2,4-pyrimidinediamine derivative) (19). The EML4-ALK C1156Y mutation can contribute to a higher resistance to PDD than those in the L1196M mutant form. It is reported that a candidate ALK inhibitor TAE684 can bind to these mutant kinases, which may have potency in overcoming the mutation-driven resistance (41). The new generation ALK inhibitors lorlatinib and brigatinib were approved in 2018 for the treatment of patients with ALK-rearranged NSCLC. Lorlatinib has been demonstrated to inhibit resistant ALK mutations, including ALK G1202R (16). However, Shaw et al. showed that an ALK L1198F mutation together with the C1156Y mutation results in the resistance of lorlatinib in a patient with metastatic ALK-rearranged NSCLC (18). However, the L1198F mutation re-sensitized crizotinib treatment of a resistant tumor. It was demonstrated that both lorlatinib and brigatinib can overcome crizotinib resistance in NSCLC patients (42, 43). Moreover, when brigatinib was combined with anti-EGFR antibody, it was effective against EGFR triple-mutant cells *in vitro* and *in vivo* (44).

## Strategies for Overcoming Mutation-Driven Resistance

Mutations in cancer therapy targets usually result in the loss of functions and the accumulation of dysfunctional proteins in tumors (45). Moreover, many mutants have oncogenic gain-of-function (GOF) activities including increased tumor proliferation, metastasis and drug resistance (46). Notably, tumor cells that receive targeted therapy may lead to an overactivation of the by-pass signaling pathways to develop resistance. In most cases, multiple alterations are observed in a resistant tumor.

Recently, many strategies dealing with mutation-driven drug resistance have been proposed and evaluated both experimentally and clinically. The traditional chemotherapy concept of “one ligand to one receptor” for a biological response is inadequate. The treatment of a particular type of cancer with the prescriptive drugs involves many special genes, interacting with their respective targets and triggering a series of biological responses. The concept of using multi-drug therapy and seeking multifunctional compounds that can efficiently interact with various targets might be feasible (47). Currently, to overcome mutation-driven drug resistance, the main strategies include: (1) the design of new mutation-targeted compounds to restore wide-type protein activities, to delete mutants or to influence downstream targets; (2) the application of combinational therapy or new compounds for multiple targeting. Here, we give some examples of how to overcome mutation-driven resistance of targeted therapy.

### Dacomitinib, an Irreversible Pan-ERBB Inhibitor, Targeting EGFR Activating Mutants

Recently, dacomitinib was approved to use for metastatic NSCLC with EGFR exon 19 deletion or exon 21 L858R substitution mutations. In a randomized, multicenter, open-label, phase III trial (ARCHER 1050), the patients with newly diagnosed advanced NSCLC and one EGFR mutation (exon 19 deletion or L858R) received a 45 mg/day dose of oral dacomitinib or a 250 mg/day gefitinib for 28 days. In the dacomitinib group, the progression-free survival (14.7 months, 95% CI 11.1–16.6) was significantly longer than that in the gefitinib group (9.2 months, 95% CI 9.1–11.0) (48). This investigation supports the dacomitinib as the first line therapy for EGFR-mutation NSCLC patients.

Dacomitinib is initially designed for irreversible pan-ERBB inhibition. As a small-molecule covalent binding inhibitor of enzymatically active HER family tyrosine kinases EGFR and HER2, it may act as a potent inhibitor of EGFR T790M mutation (49). Additionally, dacomitinib significantly inhibits both wild-type and the gefitinib-resistant ERBB2 mutation in lung cancer. Based on an in-depth investigation, dacomitinib is an effective drug that may treat NSCLC patients with a T790M-related acquired resistance to gefitinib or erlotinib (8). It has been indicated that dacomitinib significantly improves progression-free survival in EGFR-mutation NSCLC patients and is considered as a new treatment option for this population.

### Encorafenib/Binimetinib and Dabrafenib/Trametinib for Dual Inhibition of BRAF and MEK

The FDA approved dabrafenib plus trametinib for the anaplastic thyroid cancer (ATC) with BRAF-V600E mutation in May 2018, as well as for the adjuvant treatment of BRAF V600E/K-mutated melanoma in April 2018. Previous studies revealed that dabrafenib plus trametinib have shown substantial antitumor activity in patients with previously treated BRAF-V600E mutated metastatic NSCLC and untreated BRAFV-600E mutated NSCLC (50, 51). Trametinib is an orally administered MEK1/MEK2 inhibitor that suppresses RAF-dependent MEK phosphorylation and persistently inhibits phosphorylated ERK (a substrate of MEK) (52). Dabrafenib is a reversible and high-efficiency ATP-competitive inhibitor of RAF kinases, especially the mutant BRAF (53). Subbiah et al. reported that the overall response rate of dabrafenib plus trametinib applied in BRAF V600E-mutated ATC (complete reaction plus partial reaction to the best overall response) is 69% (54). In contrast to BRAF inhibitor monotherapy, it has longer progression-free survival and overall survival. Overall, the most common adverse events include fatigue, pyrexia and nausea (54), consistent with previous reports in advanced or metastatic melanoma and NSCLC (50). Dabrafenib plus trametinib is the first regimen approved to have significant clinical efficacy in BRAF V600E-mutated ATC.

In June 2018, the FDA approved the combination of BRAF inhibitor encorafenib and the MEK inhibitor binimetinib for treatment of patients with unresectable or metastatic melanoma with a BRAF-V600E or -V600K mutation. It is the third BRAF/MEK inhibitor combination approved following the dabrafenib/trametinib and vemurafenib/cobimetinib combinations (55). The main adverse events for encorafenib plus binimetinib when applied to BRAF-V600 mutant melanoma are gastrointestinal reactions, including nausea, diarrhea and vomiting. Additionally, this combination has a lower calorific value and photosensitivity than other available BRAF-MEK inhibitor combinations do (56). Considerable evidence supports that the median progression-free survival was 14.9 months with encorafenib plus binimetinib, compared with 7.3 months with vemurafenib (57). Therefore, it is an effective therapeutic option in patients with unresectable or metastatic melanoma, with a BRAF V600E or V600K mutation.

## Conclusions

In the new era of targeted therapy, treatment options are increasingly based on the precise molecular and genetic profiling of tumor cells (58). Currently, the main challenge for further novel drug development in targeted therapy is the clarification of specific molecular mechanisms underlying the varied forms of tumors in clinic. It has been acknowledged that cancer is caused by a set of driver mutations. In this regard, it is of great significance to: (1) identify and validate key mutant genes and proteins in cancers as new targets; (2) identify patients most likely and unlikely to benefit from certain targeted therapies; (3) evaluate the mechanism of mutation-driven drug resistance. In past decades, several key mutations which influence drug sensitivity have been identified in various cancers. In order to deal with mutation-driven drug resistance, new methods and drugs have been discovered and approved for clinical use (47). Even so, detailed individualized treatment strategies targeting specific tumorigenesis and drug resistant mechanisms are still required. Advances in these areas would allow for more targeted and effective therapeutic options for more cancer patients.

## Author Contributions

ML, JS, JL, YZ, JW, LQ, and QW were responsible for the review of the literature. JJ, JY, XW, QZ, CC, and ML wrote the manuscript. XW and LQ drew the Tables. XW, TY, and ZX designed the study and contributed with the valuable discussion and revision of the manuscript.

### Conflict of Interest Statement

The authors declare that the research was conducted in the absence of any commercial or financial relationships that could be construed as a potential conflict of interest.
